# A robotic object hitting task to quantify sensorimotor impairments in participants with stroke

**DOI:** 10.1186/1743-0003-11-47

**Published:** 2014-04-02

**Authors:** Kathrin Tyryshkin, Angela M Coderre, Janice I Glasgow, Troy M Herter, Stephen D Bagg, Sean P Dukelow, Stephen H Scott

**Affiliations:** 1 School of Computing, Queen’s University, Kingston, ON, Canada; 2 Centre for Neuroscience Studies, Queen’s University, Kingston, ON, Canada; 3 University of South Carolina, Columbia, SC, USA; 4 Providence Care, St. Mary’s of the Lake Hospital, Kingston, ON, Canada; 5 Hotchkiss Brain Institute, University of Calgary, Calgary, AB, Canada; 6 Department of Biomedical and Molecular Sciences, Queen’s University, Kingston, ON, Canada

**Keywords:** Stroke, Assessment, Motor skills, Robotics, Rehabilitation

## Abstract

**Background:**

Existing clinical scores of upper limb function often use observer-based ordinal scales that are subjective and commonly have floor and ceiling effects. The purpose of the present study was to develop an upper limb motor task to assess objectively the ability of participants to select and engage motor actions with both hands.

**Methods:**

A bilateral robotic system was used to quantify upper limb sensorimotor function of participants with stroke. Participants performed an object hit task that required them to hit virtual balls moving towards them in the workspace with virtual paddles attached to each hand. Task difficulty was initially low, but increased with time by increasing the speed and number of balls in the workspace. Data were collected from 262 control participants and 154 participants with recent stroke.

**Results:**

Control participants hit ~60 to 90% of the 300 balls with relatively symmetric performance for the two arms. Participants with recent stroke performed the task with most participants hitting fewer balls than 95% of healthy controls (67% of right-affected and 87% of left-affected strokes). Additionally, nearly all participants (97%) identified with visuospatial neglect hit fewer balls than healthy controls. More detailed analyses demonstrated that most participants with stroke displayed asymmetric performance between their affected and non-affected limbs with regards to number of balls hit, workspace area covered by the limb and hand speed. Inter-rater reliability of task parameters was high with half of the correlations above 0.90. Significant correlations were observed between many of the task parameters and the Functional Independence Measure and/or the Behavioural Inattention Test.

**Conclusions:**

As this object hit task requires just over two minutes to complete, it provides an objective and easy approach to quantify upper limb motor function and visuospatial skills following stroke.

## Background

The distributed nature of brain processing means that even a small stroke can impact many brain processes, necessitating a broad comprehensive approach to stroke assessment. Assessment is vital to understanding an individual’s deficits, determining their prognosis, deciding on their treatment, and monitoring their recovery. However, many challenges exist with current stroke assessment methods, particularly of sensorimotor function [1]. Most rely on observer-based ordinal scales (e.g., MRC Scale of Muscle Power [2]; Chedoke-McMaster Assessment – Impairment Scale (CMSA) [3], Fugl-Meyer [4] (FM)). Relatively course-rating scales are required to ensure reliable measurements, but often obscure the ability to quantify subtle but clinically important functional changes. Further, scales may have floor or ceiling effects (e.g., Fugl-Meyer [5,6]) and rely on criteria rather than normal-value reference standards (i.e., CMSA, FM). Finally, a comprehensive analysis of all brain faculties with standardized measures can be time consuming, and often leads to omission of assessments in clinical practice.

The need for improved assessment methods has been recognized in stroke rehabilitation [1,7-9]. Robotic technology has made a profound impact uncovering basic knowledge on quantifying motor function and learning [10,11]. Our general hypothesis is that robotics can provide a novel approach for quantifying sensory, motor and cognitive impairments associated with neurological disorders [1]. Robotics have been used to quantify sensorimotor function in individuals with stroke [12-27]. These studies demonstrate the ability to accurately and precisely quantify upper limb impairments in proprioception [12], postural control [13], motor coordination [25,26], visuomotor reactions and/or reaching performance [13,18,19,27]. Further, robots or other motion capture technologies have also been used to quantify the prevalence of sensorimotor impairments in the ipsilesional limb [13,28-33].

Here we introduce an object hitting task that quantifies simultaneous bimanual sensorimotor performance of the upper limb. The present task offers the ability to directly examine how much and how well an individual with stroke uses their more affected limb in an environment where they may choose not to do so (See also [34]). Task difficulty is also modified across time, increasing cognitive demand and visuospatial attention beyond previously-used reaching paradigms [13,16] and many simple pencil and paper tasks [35]. Lastly, the current task is quick to complete, requiring just over two minutes, while providing considerable information about participant performance. Our results highlight how many participants with stroke show impairments in task performance as compared to a large cohort of healthy control participants.

## Methods

### Participants

Participants with first clinical presentation of stroke were recruited from the stroke rehabilitation service at Providence Care, Saint Mary’s of the Lake Hospital site in Kingston, Ontario, and from the stroke units at Foothills Medical Centre and the Dr. Vernon Fanning Care Centre in Calgary, Alberta. Participants with stroke were broadly categorized into right-affected (RA) or left-affected (LA) based on the most affected side of the body. Non-disabled control participants were recruited from the communities of Kingston, Ontario and Calgary, Alberta.

Participants had 1) no significant neurological impairment (other than stroke for the stroke group), 2) were medically stable, 3) had normal or corrected to normal visual acuity, 4) had no ongoing musculoskeletal injuries of the upper limb, and 5) were able to understand the instructions of the task. The testing procedures were approved by the Research Ethics Boards of Queen’s University, Providence Care, the University of Calgary and the Dr. Vernon Fanning Care Centre. All participants gave written informed consent to be involved in this study.

### Clinical assessment

The Modified Edinburgh Handedness Inventory score [36] was used to identify handedness. For participants with missing inventory scores (n = 9), the self-reported hand dominance was used instead. Participants with mixed handedness (n = 5) were categorized as left-handed if their inventory score was below zero and right-handed otherwise.

Participants with stroke completed a number of standardized clinical assessments performed by a trained study physician or physiotherapist. Visuospatial skills were assessed with the Behavioural Inattention Test (BIT) [35], and upper limb impairment was assessed with the Chedoke-McMaster Stroke Assessment Scale – Impairment Inventory for hand and arm (CMSAh and CMSAa) [3]. Most participants with stroke were screened with the Montreal Cognitive Assessment (MoCA) [37]. Lastly, participants with stroke completed the Functional Independence Measure (FIM) [38].

### Experimental setup

**Figure 1:**
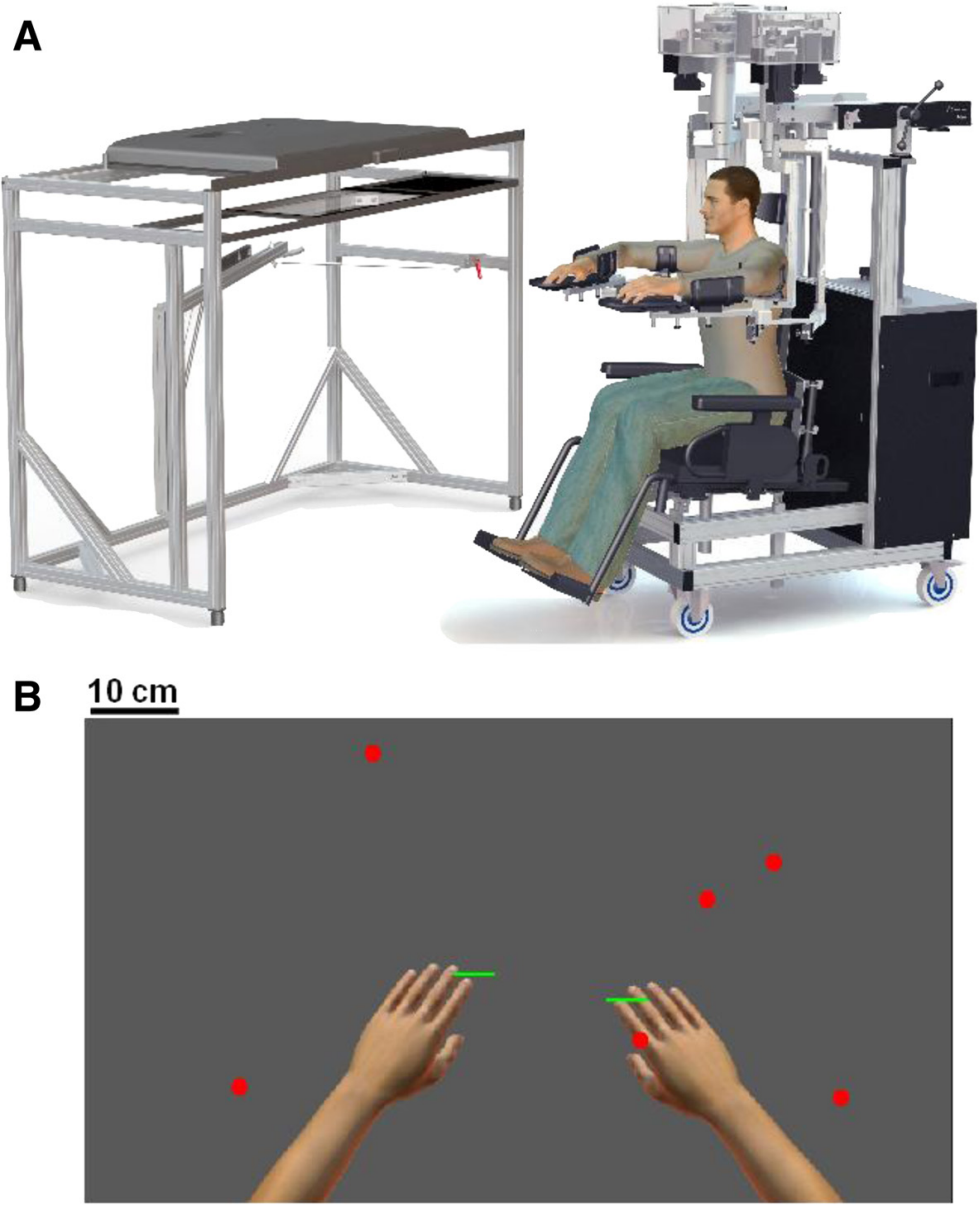
**Experimental setup. A)** The KINARM robot. Participants are seated in a wheel-chair base while their arms are resting on plastic arm troughs (one trough each for arm, forearm and hand). The setup permits movements of the arms in the horizontal plane, while providing full gravitational support for the participant's arms. The troughs are attached to an adjustable four-bar linkage. The experimenter adjusts the linkage and the troughs for each individual participant, insuring comfortable and correct position of the participant during the experiment. Visual targets and virtual paddles attached to the hands are displayed on a horizontally positioned monitor and two way mirror positioned below the monitor allows the subject to view the targets and paddles as appearing in the horizontal workspace of the participant's arms. The virtual environment is achieved by occluding direct vision of the participant's arms. **B)** A screen shot of the Object Hit task. Hand positions are displayed for illustrative purposes, but were not visible during performance of the task.

Participants underwent one robotic session using a bimanual KINARM exoskeleton robot (Figure 1A; BKIN Technologies Ltd., Kingston, Ontario). The details of the KINARM robot have been described previously [12,13,39]. Briefly, the system permits movements of the arms in the horizontal plane, while providing full gravitational support. The participants sat while their arms were attached to an adjustable four-bar linkage using plastic arm troughs (one trough each for arm, forearm and hand). The experimenter adjusted the linkage and the troughs for each individual participant ensuring free movement of the shoulder and elbow joints in the horizontal plane. Visual targets were projected in the plane of the participant’s arms using a two-way mirror. Direct vision of the participant’s arms was occluded. It takes 10 to 15 minutes to setup and calibrate a subject in the robotic system. Operators trained to use the system normally receive ~10 hours of formal training on how to setup subjects and use the software, including calibrating 5 to 10 subjects.

### Experimental task

**Figure 2:**
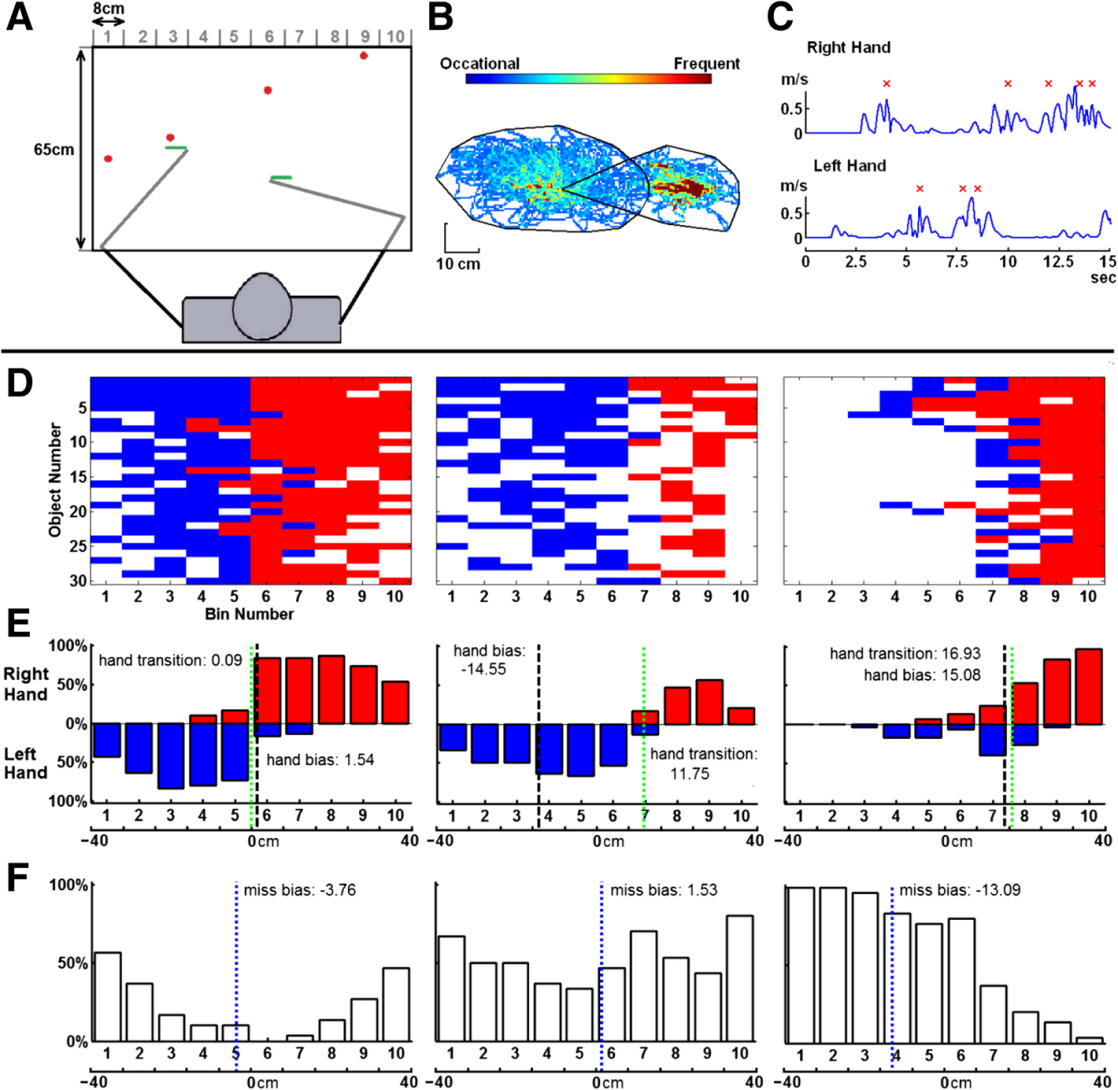
**Object hitting task and example performance. A)** Workspace for task. Balls move towards the participant from one of 10 bins (bins not displayed to participant). **B)** Hand trajectories for the left and right hands during the task of an exemplar, right-affected participant with stroke. The black lines define hand movement area for each hand. **C)** Hand speed for a few seconds during the task. Successful ball hits are marked with a red x. **D)** Performance grids for three exemplar participants (left: control, middle, right-affected participant, right, left-affected participant with visuospatial neglect). The x-axis represents the 10 bins from which balls are dropped. The y-axis corresponds to the 30 random blocks, where the top row corresponds to the first random block (easiest) and the last row corresponds to the last block. Successful hits made with the right hand are colored red, hits with the left hand are coloured blue and misses are white. **E)** Hits distribution of right (red) and left (blue) hands. Hand transition is shown with the green line. **F)** Distribution of misses with miss bias shown with the blue line.

Participants were instructed to use both hands, represented as 5 cm wide green paddles, to hit 2 cm diameter virtual red balls that moved towards them (see Figures 1B, 2A-C). The objective of the task was to hit away as many balls as possible. The balls appear at random from 10 different bins (located 8 cm apart, but the bins themselves are not displayed), with a total of 30 balls released from each bin. All 10 bins release a ball in random order before a bin is reused. The number of balls that appear on the screen at a given moment and their speed increase throughout the task such that one single, slow-moving (~10 cm/s) ball is visible on the screen at the beginning of the task and a maximum of 16 fast-moving (~50 cm/s) balls are present towards the end of the task. Objects begin to drop at a rate of 0.5 + 0.025 * Time (in units of seconds) and drop speed is randomly selected between 50 to 100% of maximum drop speed, where maximum drop speed is 15 + 3 * Time (in units of cm/s). A total of 300 balls fall in just over 2 minutes. The robot generated a 50 ms force pulse, based on the ball’s acceleration following contact with the paddle, to simulate feedback of object contact. Positions and velocities of the hands and balls are recorded with a sampling frequency of 200 Hz.

### Data analysis

Data analysis was performed using MATLAB (Mathwork, Inc., Massachusetts, USA). Twelve parameters were developed to quantify task performance.

A. Global Performance

*Hit percentage:* a successful hit occurs when the ball is hit and leaves the display area at the top or sides of the workspace (If ball hit softly, the ball fades away after 4.5 seconds. Its direction of movement defines success or failure). When a ball is hit multiple times, the first hit indicates which hand was used and the last hit indicates if it was successful.

B. Spatial and Temporal Performance

*Miss bias:* quantifies whether there was a spatial bias in the position of balls missed in the workspace. This value (the weighted mean) is computed as the sum of the number of misses in each bin (m) multiplied by the bins position in the frontal plane (x), and then dividing by the total number of misses (sum(mx)/sum(m)).

*Hand transition:* the line in the workspace where the participant's preference switches from one hand to the other. It is computed by taking the mean of two values: the right hand and the left hand weighted means of hit distributions. The weighted mean (See Miss bias) of hit distributions for each hand are calculated independently using a subset of bins, including only those where both hands made hits (overlapping bins) plus one additional bin on each side of the overlapping bins. In the special case where no overlap occurs, the subset of bins used includes the rightmost bin in which hits were made by the left hand and the left-most bin where hits were made with the right hand.

*Median error (ball%):* the point in the task where the participant missed half of the balls that they missed in the entire task as a percentage of the total number of balls. Higher scores reflect that the participant performed relatively well when the task was easy and failed predominantly at the end of the task when the task difficulty was greatest.

C. Motor Performance

a) Right and Left hands

*Movement area:* captures the area of space used by each hand during the task. It is computed as the area of the convex hull [40], which is a convex polygon that captures the boundaries of the movement trajectories of each hand (Figure 2B). It is computed for each hand separately.

*Mean hand speed:* the average hand speed during the task. Encoders attached to the motors quantify joint motion. Hand speed is calculated from joint velocities measured by the KINARM robot and the length of the arm segments.

b) Bimanual

*Hand bias hits:* identifies the relative hand use. It is the normalized difference between the total number of hits with right (RH) and left (LH) hands (RH hits – LH hits) / (RH hits + LH hits).

*Hand selection overlap:* quantifies whether both hands share the workspace. The score is computed by counting the number of times that two successive balls hit from a given bin use different hands (i.e. ball hit by left hand and the next successful ball hit from that bin was with the right hand, or vice versa) divided by the total number of hits.

*Hand movement bias area:* quantifies differences in the size of the workspace used by each hand. It is the normalized difference between hand movement areas of the left and right hands (area of the RH – area of the LH)/(area of the RH + area of the LH).

*Hand bias speed:* quantifies differences in hand speed. It is the normalized difference between mean hand speeds of the left and right hands (mean hand speed of the RH – mean hand speed of the LH)/(mean hand speed of the RH + mean hand speed of the LH).

### Statistical analysis and data processing

Hand speeds were filtered using a sixth-order double-pass Butterworth filter with cutoff frequency of 10 Hz. Values for miss bias, hand transition, hand bias hits, total hand area, hand movement area bias, mean hand speed and hand bias speed were flipped for left-handed participants. Values for miss bias, hand transition and hand bias hits were transformed to -40 to +40 to correspond to the horizontal dimension of the screen in units of centimeters.

Effects of age, sex and test-arm (dominant or non-dominant) were examined in our cohort of control participants as follows. First, univariate, simple, linear regressions were performed to quantify age-related effects on each parameter. In the analysis each parameter (response variable) was studied in relation to subject age (predictor variable). The regression coefficients were estimated using non-least-squares linear method (robustfit, MATLAB function). The residuals from the regression were tested to verify that they were normally distributed. If not normal, we transformed the data using logarthimic, square root or inverse transforms. Consequently, a test was done to detect for effects of sex (males versus females) and test-arm on the age-tested parameters (two-sample Kolmogorov-Smirnov test, p < 0.01). If differences were found, data was grouped by sex or test-arm, and regression analysis was again performed.

Impairments in a given parameter were identified by comparing individual participants relative to <5% or >95% of the healthy control population considering age, sex and test-arm. We used <2.5% and >97.5% thresholds when poor performance could reflect either values above or below healthy control performance. The use of an upper and/or lower bound for each parameter is listed in Table 1.

**Table 1:** Performance of participants with stroke

**Parameters**	**Cut off range (%)**	**LA stroke (%)**	**RA stroke (%)**	**Spatial neglect LA(%)**	**Spatial neglect RA(%)**	**Inter rater (r)**	**Correlations**						
							**MoCA**	**FIMm**		**FIMt**		**BIT**	
**Global performance**													
**Hit percentage**	<5	87	67	100	80	0.97	**0.37**	**0.58**		**0.62**		**0.55**	
**Spatial and Temporal Performance**													
**Miss bias**	<2.5, >97.5	11	6	24	0	0.73	-0.01	0.21		**0.21**		**0.21**	
**Hand transition**	<2.5, >97.5	42	38	59	60	0.51	-0.12	-0.07		-0.08		-0.06	
**Median error**	<5	73	44	97	60	0.84	**0.35**	**0.58**		**0.61**		**0.50**	
**Motor Performance**													
**Hand bias (hits)**	<2.5, >97.5	73	59	90	100	0.94	0.10	**-0.21**		**-0.22**		-0.20	
**Hand selection overlap**	<5	33	19	48	20	0.53	0.07	**0.37**		**0.33**		0.20	
**Total hand bias area**	<2.5, >97.5	55	43	79	60	0.92	0.15	**-0.29**		**-0.28**		**-0.30**	
**Hand bias speed**	<2.5, >97.5	67	54	93	80	0.98	0.14	**-0.30**		**-0.30**		**-0.31**	
**Total hand area DH**	<5	16	38	28	60	0.69	0.08	**0.58**		**0.57**		**0.32**	
**Total hand area NDH**	<5	52	17	69	60	0.86							
**Hand speed DH**	<5	41	44	76	60	0.90	0.14	**0.59**		**0.60**		**0.40**	
**Hand speed NDH**	<5	73	16	93	60	0.91							
**Overall Participants Performance (# of Failed Parameters)**													
**# parameters**	**0**	**1**	**2**	**3**	**4**	**5**	**6**	**7**	**8**	**9**	**10**	**11**	**12**
**Controls**	175	45	26	8	3	3	1	1	0	0	0	0	0
**LA stroke**	6	7	3	1	5	10	11	8	19	10	6	5	0
**RA stroke**	7	10	5	5	5	6	5	6	5	7	1	1	0
**Spatial neglect LA**	0	0	0	0	0	2	2	2	9	5	4	5	0
**Spatial neglect RA**	0	0	0	1	0	1	0	0	1	1	1	0	0

*Abbreviations:RH*, Right hand; *LH*, Left hand; *RA*, Right affected participant; *LA*, Left affected stroke; *MoCA*, Montreal Cognitive Assessment; *FIM*, Functional Independence Measure, m and t after FIM denote motor and total score, respectively; *BIT*, Behavioural Inattention Test.

Values in bold indicate p-value < 0.01.

Percentages for stroke group reflect performance above and/or below bounds defined by the healthy population (defined in column: Cut off range (%). Inter-rater values reflect performance assessed in two separate sessions performed by different operators. Last 4 columns display correlations between each parameter and MoCA, FIM and BIT clinical scores. Bottom 5 rows display number of task parameters failed by each group.

## Results

### Participant pool

Table 2 shows the demographic and clinical data from 154 stroke (91 left- and 63 right-affected) and 262 control participants. The majority of the control participants were right-hand dominant. The majority of the participants with stroke had ischemic stroke (83%). There were marginal differences between left- and right-affected participants (two-sided rank sum test) on the FIM (total, p = 0.04; motor, p = 0.02), whereas no differences were observed for the MoCA (p = 0.2) scores. Thirty-four out of 154 participants with stroke had visuospatial neglect (BIT score <130, 29 left- and 5 right-affected).

**Table 2:** Demographic and clinical data

**Measure**	**Group**				
	**Left-affected participants (n = 91)**		**Right-affected participants (n = 63)**		**Control participants (n = 262)**
Age^a^	61(20, 89)		62(21, 86)		54.5(20, 89)
Sex (M/F)	62/29		38/25		134/128
Handedness(R/L/M)	86/4/1		54/5/4		239/23/0
Type of stroke(I/H)	74/17		54/9		-
Stroke location (C/SC/C + SC/B/Cr/Cr + B)^b^	36/24/23/4/3/1		28/20/9/6/0/0		-
Days since stroke^a^	15(2, 86)		21(2, 85)		-
BIT^a^	139(51, 146)		141(58, 146)		-
MoCA^a,c^	24(13, 30)		23(8, 29)		-
FIM motor score^a^	70(17, 91)		79(29, 91)		-
FIM total score^a^	100(35, 126)		107(43, 126)		-
MAS left^d,e^	0.456(0, 3)		0.016(0, 1)		-
MAS right^d,f^	0.056(0, 1)		0.429(0, 3)		-
Limb	Unaffected	Affected	Unaffected	Affected	-
CMSAa score^g^	[0,0,0,0,7,23,61]	[14,12,11,3,19,11,21]	[0,0,0,0,2,15,46]	[6-8,3,8,9,22]	-
CMSAh score^g^	[0,0,0,0,0,28,63]	[15,9,6,6,21,22,12]	[0,0,0,0,2,23,38]	[6,5,5,5,12,12,18]	-

*Abbreviations:M/F*, Male/female; *R/L/M*, Right/left/mixed; *I/H*, Ischemic/hemorrhagic; *BIT*, Behavioural Inattention Test; Mini-Mental Status Exam; MoCA, Montreal Cognitive Assessment; FIM, Functional Independence Measure; MAS, Modified Ashworth Score; CMSAh, Chedoke-McMaster Stroke Assessment – Impairment Scale hand; CMSAa, Chedoke-McMaster Stroke Assessment - Impairment Scale arm.

^a^median value, minimum and maximum values in parentheses; ^b^C/SC/C + SC/B/Cr/CR + B) represents: cortical/subcortical/cortical + subcortical/brainstem/cerebellar/ cerebellar + brainstem lesion location; ^c^assessed in n = 83 left affected, 56 right affected; ^d^mean value, minimum and maximum values in parentheses; ^e^n = 86 left affected, n = 61 right affected; ^f^n = 90 left affected, n = 56 right affected; ^g^[n_1_, n_2_, n_3_, n_4_, n_5_, n_6_, n_7_] represents number of participants with CMSA scores of [1-7].

### Individual subject exemplars

Figure 2D-F highlight exemplar participants including a healthy control (left panel, 1D), a right-affected participant (middle panel, 2D) and a left-affected participant with visuospatial neglect (right panel, 2D). The control participant displays many more hits than misses, with misses localized mostly on the far left and right sides, and occurring more frequently towards the end of the task. The control has a hand bias of 1.54 cm and hand transition at 0.09 cm from the midline, indicating equal use of both limbs with the transition in hand-use in the middle of the workspace (Figure 2E). The miss bias for the control participant (Figure 2F) is slightly shifted to the left (-3.76 cm) indicating slightly more misses on the left side for this right-hand dominant participant.

The right-affected participant with stroke clearly uses the left more than the right hand (hand bias score = -14.55 cm) and hand transition is 11.75 cm to the right indicating the left hand was used to hit balls for most of the workspace (Figure 2D-F, middle panels). In contrast, miss bias is near the center (1.53 cm), as balls were missed nearly equally on both sides of the workspace.

Performance by the left-affected participant with visuospatial neglect (BIT score = 105), is clearly different than the participant without visuospatial neglect displayed in the middle panels (right versus middle panels in Figure 2D-F). Not surprisingly, this participant hit more balls with their right hand (hand bias = 15.08 cm), but hand transition was shifted to the right side (16.93 cm). Most balls were missed on the left side of the workspace (miss bias = -13.09 cm), particularly at the end of the task.

### Performance of healthy control participants

Regression analyses identified which task parameters were affected by participant age considering possible influences of sex and hand (Table 3). Sex and age both influenced two parameters: hit percentage (males and females) and median error (males and females). Handedness (dominant versus non-dominant) did not influence any of the parameters. Percentiles (1, 2.5, 5, 25, 50, 75, 95, 97.5, 99) were obtained for each parameter based on healthy subject performance. In cases, where there were regressions, the percentiles reflect residuals relative to the linear regression. For parameters that have no age effect, subjects were identified as impaired if their parameter values were above and/or below a specific percentile, as defined by the cut-off range given in Table 1. For parameters with an age effect, the threshold to define impairment was (age*slope) + intercept + percentile.

**Table 3:** Model fits and percentiles for the object hit task parameters

**Group**	**Parameters**	**Model fit**		**Percentiles**								
		**Intercept**	**Slope**	**1**	**2.5**	**5**	**25**	**50**	**75**	**95**	**97.5**	**99**
**All**	*MB (cm)	-	-	-15.19	-10.66	-9.17	-4.12	-0.79	2.38	7.69	9.67	13.10
	*HT (cm)	-	-	-6.91	-6.51	-5.99	-3.03	-0.87	0.52	3.27	3.76	4.69
	*HBH (cm)	-	-	-5.07	-3.63	-2.17	0.54	2.11	3.87	6.09	7.19	8.63
	*HSO	-	-	0.04	0.06	0.06	0.09	0.12	0.15	0.20	0.23	0.24
	*THA_DH	-	-	0.07	0.08	0.09	0.11	0.13	0.15	0.19	0.20	0.22
	*THA_NDH	-	-	0.07	0.08	0.09	0.11	0.13	0.15	0.18	0.20	0.20
	*THBA	-	-	-0.20	-0.15	-0.13	-0.04	0.01	0.07	0.13	0.16	0.20
	*HS_DH (m/s)	-	-	0.15	0.18	0.20	0.25	0.29	0.34	0.41	0.44	0.48
	*HS_NDH (m/s)	-	-	0.16	0.18	0.20	0.25	0.29	0.34	0.42	0.45	0.49
	*HBS	-	-	-0.19	-0.14	-0.11	-0.05	-0.01	0.04	0.11	0.12	0.21
**Male**	**HP (%)	93.7	-0.32	-23.33	-16.61	-15.04	-5.42	0.67	5.42	12.32	14.18	16.01
	**ME	80.3	-0.16	-11.12	-10.18	-9.42	-2.97	-0.31	3.56	10.20	11.50	13.74
**Female**	**HP (%)	83.8	-0.25	-24.60	-20.08	-17.34	-5.67	-0.03	4.76	13.16	13.88	17.29
	**ME	73.8	-0.09	-10.31	-9.31	-7.95	-3.31	-0.33	3.92	7.15	9.21	11.38

*Abbreviations: MB*, Miss bias; *HT*, Hand transition; *HBH*, Hand bias of hits; *HSO*, Hand selection overlap; *THA_DH*, Total hand area dominant hand; *THA_NDH*, Total hand area non-dominant hand; *THBA*, Total hand bias area; *HS_DH*, Hand speed dominant hand; *HS_NDH*, Hand speed non-dominant hand; *HBS*, Hand bias speed; *ME*, Median error, *HP*, Hit percentage.

*y = percentile.

**y = (slope*age) + intercept + percentile.

### Performance of participants with stroke

Table 1 displays the percentage of participants with stroke that were identified as different from controls. Parameters that identified the largest number of participants with stroke as impaired were: hit percentage (87% of left- and 67% of right-affected), hand bias hits (73% of left- and 59% of right-affected), and hand bias speed (67% of left- and 54% of right-affected). The fact that total hit percentage identified the largest number of participants with stroke as different from controls should not be surprising as overall task performance can be influenced by a variety of sensory, motor and cognitive impairments. Most participants with visuospatial neglect (29 left-affected and 5 right-affected) were identified as different from controls, as all but one hit fewer balls and displayed a greater hand bias than 95% of controls.

**Figure 3:**
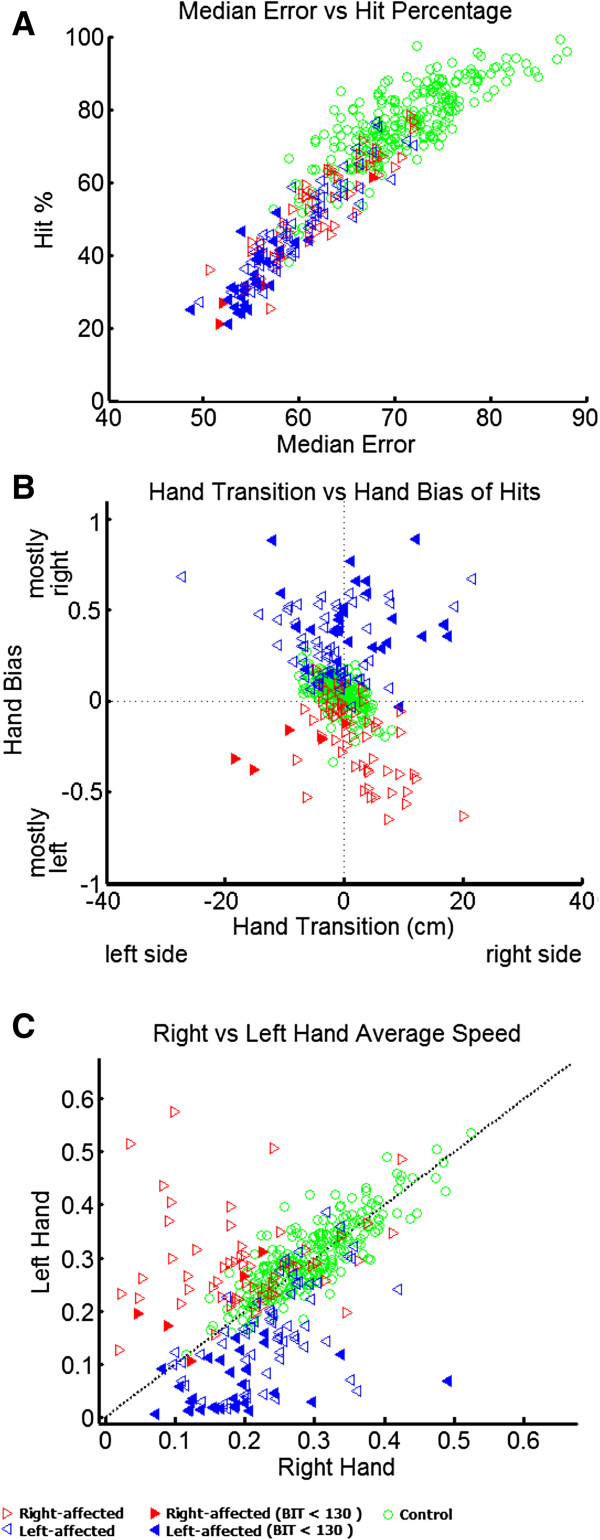
**Participant performance for selected parameters. A)** Median error versus hit percentage **B)** Hand transition versus hand bias of hits. **C)** Average speed for the right and left hands. Control participants are represented as green open circles, right-affected participants are the red, right-pointing triangles and left-affected participants are shown as blue, left-pointing triangles. Participants with visuospatial neglect are denoted by filled triangles.

Figure 3 presents performance of all participants for a number of selected parameters. Figure 3A displays median error versus hit percentage. The values of both parameters are much lower for participants with stroke as compared to controls, especially for participants with visuospatial neglect (filled triangles). Participants with stroke tend to miss balls earlier in the task than control participants. In the extreme case, some participants with stroke have median scores near 0.5, indicating they missed proportionally the same number of balls at the beginning of the task when the task is relatively easy as at the end of the task when many fast moving balls are present.

Figure 3B displays hand transition versus hand bias of hits. Control participants are generally clustered around zero for both scores denoting relatively equal use of both hands to hit objects and the transition from using one limb versus the other occurred near the midline. As expected, left-affected participants always hit more balls with their right hand, whereas right-affected participants use the left hand more. Hand transition measures were more complex and variable. Participants in the upper left and lower right quadrants denote an expected trade-off between hand use and the transition point in the workspace for using the two hands. Most right-affected participants displayed this pattern with the left hand hitting more balls and the transition between using the two hands is on the right side of the body (exemplified by the middle panel in Figure 2D). This expected trade-off was not as common in left-affected participants who often displayed greater use with the right hand (expected), but the transition from using the left and right hands was also shifted to the right (unexpected). These unexpected patterns of behavior were commonly observed for participants with BIT scores below 130 (filled triangles), although some other participants with stroke also displayed this pattern.

Figure 3C displays the average speed for the left and right hands. There was a broad range of speeds utilized by control participants, ranging from 0.12 m/sec to 0.54 m/sec. However, left and right hand speeds for control participants were highly correlated (r = 0.83), indicating that if they moved quickly/slowly with one limb they also moved the other limb quickly/slowly. In contrast, participants with stroke commonly displayed asymmetric hand speeds with the affected limb commonly moving much slower than their less affected limb. This was particularly common for left-affected participants with visuospatial neglect (filled blue triangles).

The number of parameters identified as different from the control population is displayed at the bottom of Table 1. More than half of control participants (67%) passed all the parameters, with 3% of control participants identified as outside the 5% or 95% range (or 2.5% and 97.5% range when necessary) for 4 or more parameters. In contrast, 57% of right-affected and 81% of left-affected participants were identified as impaired on 4 or more parameters. Moreover, 53% of left-affected participants performed very poorly in the task as they were identified as impaired for 7 parameters or more. On the other hand, right-affected participants were more uniformly distributed with regards to the number of parameters that were different from controls. All but one participant with visuospatial neglect was identified as impaired on 4 or more parameters and over 82% of visuospatial neglect participants failed on 7 or more parameters.

### Inter-rater reliability

We examined inter-rater reliability by retesting 31 participants (11 control and 20 stroke). During each retest a different operator performed the KINARM setup. Participants were retested within 2 days of the original assessment and often on the same day. Correlations were generally very high across sessions with half the values above 0.90 (Table 1). Hand speed bias displayed the highest correlation at 0.98 with hit percentage also attaining a high correlation (0.97).

### Comparison between robotic and clinical measures

Task parameters were compared to the MoCA, FIM and BIT scores collected for the participants with stroke (Table 1). Almost all parameters displayed significant Spearman correlations with FIM total and FIM motor (Table 1). Most task parameters significantly correlated with BIT scores, notably hit percentage and median error.

## Discussion

The present study describes the use of a rapid, bimanual motor task to quantify sensorimotor capabilities of participants with stroke. Many of the task parameters correlated with traditional clinical measures and most parameters had very high inter-rater reliability. Importantly, participants reported the task was enjoyable to perform with simple instructions and can be completed in just over two minutes. Further, the task has effectively no floor or ceiling effects.

### Participant-based assessment

Many studies highlight differences between motor function of participants with stroke as compared to healthy controls [14,22,26,41-46], but these studies are based on group-level comparisons. In general, such studies match each participant with stroke to a healthy control participant with the same age and sex. We could have computed group level effects, and likely would have found differences between the two groups for all parameters, except perhaps Miss Bias. Unfortunately this approach cannot quantify whether a specific participant is significantly different from healthy controls.

For most clinicians, it is usually far more important to understand how each individual differs from “normal”. Thus, we quantified control performance of a large cohort of male and female participants aged 20 to 89 with no known neurological disorders. We then identified whether sex, age and test-arm influenced various task parameters and developed regression models to consider these attributes. This approach allowed us to identify whether or not each participant fell within the typical range for healthy controls considering these attributes. While uncommon for assessing sensorimotor control, such an approach is common for other clinical laboratory tests (eg. Blood tests, nerve conduction studies) used to determine an individual’s prognosis and plan for treatment. With the object hitting task, we can also obtain information about use of the more affected arm after stroke, in a situation where the participant can choose (unconsciously or consciously) to use the less affected arm. Potentially, this has important implications on determining whether rehabilitation of the affected arm is carrying over into more complex tasks. Further, it quantifies real-time visuospatial skills in a more complex, interactive environment than possible with simple tasks such as line bisection.

### Inter-limb differences

A previous study noted that asymmetries in motor performance between the arms provide a useful way for quantifying small, but significant impairments in performance [13]. The present study also found measures of asymmetry was useful for identifying impairments. After total hit percentage, the two best parameters for separating participants with stroke from healthy controls were hand bias hits and hand bias speed. Both parameters provide a relative comparison on the use of each limb during the task, with fewer hits and slower speeds normally observed for the affected limb. These comparative scores can identify participants that display hand speeds within the typical range for healthy controls, but have asymmetric performance between the limbs. These comparative parameters take advantage of the fact that healthy performance is usually relatively symmetric, whereas a hallmark of subjects with stroke is asymmetric behaviour.

It is important to recognize that the presence of asymmetries in limb motor function likely lead to a preference for using the unimpaired rather than the impaired limb for daily activities. An important aspect of rehabilitation is to minimize this learned disuse. With regards to assessing this asymmetry in the use of the limbs, a recent study introduced a reaching task in which the participants could select which limb they prefer to reach to objects located in different directions and distances [34]. They found that subjects with stroke usually reached with their unimpaired limb for the majority of the workspace. Although subjects received gravitational support in our study, the rapid nature of the object hitting task allowed us to quantify quickly subject limb choice under varying levels of task difficulty and increasing cognitive demand and visuospatial attention.

### Left- versus right-affected participants

Our robot-based assessment and traditional measures of disability (FIM) and impairment (CMSA) tended to identify greater disabilities/impairments in left-affected stroke subjects. Participants were recruited in the hospital environment and inclusion criteria were designed to examine those with a wide variety of lesion locations and severities, reflective of clinical practice in stroke rehabilitation. There are several factors that may explain why impairments were more common in left-affected participants. First, the need to understand task instructions may have reduced the number of severely impaired right-affected participants due to receptive aphasia. Second, recent work highlights a number of hemispheric specializations associated with visuomotor performance [46-50]. In particular, greater deficits for left-affected participants may reflect the dominant role of the right hemisphere in spatial attention [51-54]. Visuospatial neglect was far more common in left-affected participants in our cohort. As the object hitting task requires good visuomotor skills, it is not surprising that greater impairments were observed for left-affected participants.

On its own, the object hitting task performed on this robotic technology is clearly not cost effective in clinical practise. However, the value of such robotic technology is the ability to automate and perform a broad range of tasks to quantify sensory, motor and cognitive processes [1]. We have developed other robot-based tasks, including a visual-guided reaching task and a limb matching task to assess other aspects of sensorimotor function, and these tasks have been used to study impairments in subjects with stroke [12,13,55], traumatic brain injury [56], and fetal alcohol spectrum disorder in children [57]. As more tasks are developed and more patient groups are examined, these advanced technologies may provide not only important information for clinical research on neurological disorders, but also prove useful in the clinical environment.

## Conclusions

The present study used an object hitting task to quantify sensorimotor impairments in individual participants with stroke. Twelve parameters were used to quantify participant performance including overall task success, spatial and temporal aspects of behaviour, and the capabilities of each arm. We found that 87% of left-affected and 67% of right-affected participants were identified as impaired in overall task success. Virtually all participants with visuospatial neglect were identified as impaired in the task. Inter-rater reliability was quite high for most parameters illustrating the ability of using robotic technology to objectively and reliably assess participants’ performance.

## Competing interests

SHS is co-founder and scientific officer of BKIN Technologies, the company which commercializes the KINARM robotic device. The other authors KT, AC, JIG, TMH, SDB and SPD have no competing interests to declare.

## Authors’ contributions

KT analyzed the collected data, designed and developed the parameters, carried out the statistical analysis, drafted the manuscript. AC collected some of the experimental data. JIG was involved in the design of the study and data analysis helped to draft the manuscript. TMH was involved in data analysis. SDB was involved in patient recruitment. SPD was involved in patient recruitment, participated in data analysis and drafting the manuscript. SHS participated in the design of the task, data analysis and drafting the manuscript. All authors read and approved the final manuscript.
